# Supplementary material for: YAP regulates cell size and growth dynamics via non-cell autonomous mediators
Source: eLife. 2020 Jan 9;9:e53404. doi: 10.7554/eLife.53404 (PMC6989120; doi:10.7554/eLife.53404)
Supplement: Transparent reporting form [file elife-53404-transrepform.docx]

***eLife’s* transparent reporting form**

We encourage authors to provide detailed information *within their submission* to facilitate the interpretation and replication of experiments. Authors can upload supporting documentation to indicate the use of appropriate reporting guidelines for health-related research (see [EQUATOR Network](http://www.equator-network.org/%20)), life science research (see the [BioSharing Information Resource](https://biosharing.org/)), or the [ARRIVE guidelines](http://www.plosbiology.org/article/info:doi/10.1371/journal.pbio.1000412) for reporting work involving animal research. Where applicable, authors should refer to any relevant reporting standards documents in this form.

If you have any questions, please consult our Journal Policies and/or contact us: [editorial@elifesciences.org](mailto:editorial@elifesciences.org).

**Sample-size estimation**

- You should state whether an appropriate sample size was computed when the study was being designed
- You should state the statistical method of sample size computation and any required assumptions
- If no explicit power analysis was used, you should describe how you decided what sample (replicate) size (number) to use

Please outline where this information can be found within the submission (e.g., sections or figure legends), or explain why this information doesn’t apply to your submission:

We did not report the use of any animals for this manuscript. We used a well-established cell line (HEK293 and Flipin-Trex-293) which, in principle, makes the number of replicates per experiment none limiting. Technically, the number of replicates per experiment were determined based on the number of conditions compared and the format of the plates being used. For example, when comparing two cell lines under 10 conditions, the maximum number of replicates possible per cell line on a 96-well plate is 6, but usually 5 were used because of edge-effects which often prevent the use of the outermost wells. If the experiment is done with two cell lines on a 6 well plate, the number of replicates we are limited to is 3, and edge-effects are considered to be uniform across replicates. Assays done on 10 cm dishes were done with single replicates for each condition, and repeated independently on different days. In this case, a quantitative comparison across days is not possible and the data depicted is from a single experiment without any statistics. However, a qualitative comparison of the relative change between samples prepped and measured on the same day is done for the experiments performed on different days to confirm reproducibility.

This information is included in the figure legends for most data shown. For the RNAseq and proteomics data, it is included in the methods section.

**Replicates**

- You should report how often each experiment was performed
- You should include a definition of biological versus technical replication
- The data obtained should be provided and sufficient information should be provided to indicate the number of independent biological and/or technical replicates
- If you encountered any outliers, you should describe how these were handled
- Criteria for exclusion/inclusion of data should be clearly stated
- High-throughput sequence data should be uploaded before submission, with a private link for reviewers provided (these are available from both GEO and ArrayExpress)

Please outline where this information can be found within the submission (e.g., sections or figure legends), or explain why this information doesn’t apply to your submission:

Replicates are mentioned in the figure legends, and for the high-throughput analysis in the methods sections.

RNA-seq data has been deposited in GEO under accession number: GSE123296.

The dataset is not yet public but can be accessed by reviewers at:  <https://www.ncbi.nlm.nih.gov/geo/query/acc.cgi?acc=GSE123296>,

using the the token: onmvskyafvgbxer

**Statistical reporting**

- Statistical analysis methods should be described and justified
- Raw data should be presented in figures whenever informative to do so (typically when N per group is less than 10)
- For each experiment, you should identify the statistical tests used, exact values of N, definitions of center, methods of multiple test correction, and dispersion and precision measures (e.g., mean, median, SD, SEM, confidence intervals; and, for the major substantive results, a measure of effect size (e.g., Pearson's r, Cohen's d)
- Report exact p-values wherever possible alongside the summary statistics and 95% confidence intervals. These should be reported for all key questions and not only when the p-value is less than 0.05.

Please outline where this information can be found within the submission (e.g., sections or figure legends), or explain why this information doesn’t apply to your submission:

Any statistics are reported within the figure legends of the corresponding figure. For the high-throughput data, how the statistics were calculated is within the methods section, and the mean values and only the significantly changed transcripts are reported in Supplementary File 1.

(For large datasets, or papers with a very large number of statistical tests, you may upload a single table file with tests, Ns, etc., with reference to sections in the manuscript.)

**Group allocation**

- Indicate how samples were allocated into experimental groups (in the case of clinical studies, please specify allocation to treatment method); if randomization was used, please also state if restricted randomization was applied
- Indicate if masking was used during group allocation, data collection and/or data analysis

Please outline where this information can be found within the submission (e.g., sections or figure legends), or explain why this information doesn’t apply to your submission:

Samples were grouped together when treated similarly, usually grouping is done among biological replicates collected on the same day. Qualitative comparisons were done across experiments but no grouping was performed.

**Additional data files (“source data”)**

- We encourage you to upload relevant additional data files, such as numerical data that are represented as a graph in a figure, or as a summary table
- Where provided, these should be in the most useful format, and they can be uploaded as “Source data” files linked to a main figure or table
- Include model definition files including the full list of parameters used
- Include code used for data analysis (e.g., R, MatLab)
- Avoid stating that data files are “available upon request”

Please indicate the figures or tables for which source data files have been provided:

Figure 3, which includes RNA-seq and Mass spec data is supported by data in supplementary File 1. RNA-seq raw data is deposited in GEO (see above).
